# Synthesis and crystal structure of silicon pernitride SiN_2_ at 140 GPa

**DOI:** 10.1107/S2056989023008058

**Published:** 2023-09-19

**Authors:** Pascal L. Jurzick, Georg Krach, Lukas Brüning, Wolfgang Schnick, Maxim Bykov

**Affiliations:** a Institute of Inorganic Chemistry, University of Cologne, Greinstrasse 6, 50939 Cologne, Germany; b Department of Chemistry, University of Munich (LMU), Butenandtstrasse 5-13 (D), 81377 Munich, Germany; Vienna University of Technology, Austria

**Keywords:** nitrides, pernitrides, silicon nitrides, high pressure, crystal structure

## Abstract

Silicon pernitride, SiN_2_, was synthesized from the elements at 140 GPa in a laser-heated diamond anvil cell. Its crystal structure was solved and refined by means of synchrotron-based single-crystal X-ray diffraction data. The title compound crystallizes in the pyrite structure type (space group *Pa*  , No. 205). The Si atom occupies a site with multiplicity 4 (Wyckoff letter *b*, site symmetry .  .), while the N atom is located on a site with multiplicity 8 (Wyckoff letter *c*, site symmetry .3.). The crystal structure of SiN_2_ is comprised of slightly distorted [SiN_6_] octa­hedra inter­connected with each other by sharing vertices. Crystal chemical analysis of bond lengths suggests that Si has a formal oxidation state of +IV, while nitro­gen forms pernitride anions (N—N)^4–^.

## Abstract 2

SiN_2_ was synthesized from the elements at 140 GPa in a laser-heated diamond anvil cell. Crystal-structure determination (single-crystal synchrotron X-ray data) revealed that the title compound crystallizes in the pyrite structure type (space group *Pa* 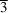 ).

## 1. Chemical context

Nitro­gen-rich materials have gained a lot of attention due to their diverse properties such as high hardness, incompressibility (Young *et al.*, 2006 ▸; Bykov *et al.* 2019*a* ▸) and high energy density (Bykov *et al.*, 2021 ▸; Wang *et al.*, 2022 ▸). Among these, binary high-pressure nitrides of group 14 elements are of particular inter­est, as they exhibit remarkable elastic and electronic properties compared to their ambient-pressure counterparts. In particular, cubic silicon nitride *γ*-Si_3_N_4_, synthesized from the elements at about 15 GPa, is significantly more incompressible than the ambient-pressure *α*- and *β*-polymorphs (Zerr *et al.*, 1999 ▸). Recently Niwa *et al.* (2017 ▸) have synthesized pernitrides of group 14 elements (SiN_2_, SnN_2_ and GeN_2_) by using laser-heated diamond anvil cells at pressures above 60 GPa. The crystal structures of GeN_2_ and SnN_2_ were solved and refined against powder X-ray diffraction data. However, the weak X-ray powder pattern of SiN_2_ only allowed the suggestion that SiN_2_ crystallizes in the pyrite structure type, while no structure refinement was performed.

In this work, we synthesized SiN_2_ from the elements at pressures of 140 GPa and examined it by means of synchrotron single-crystal X-ray diffraction in order to solve and refine its crystal structure.

## 2. Structural commentary

**Figure 1:**
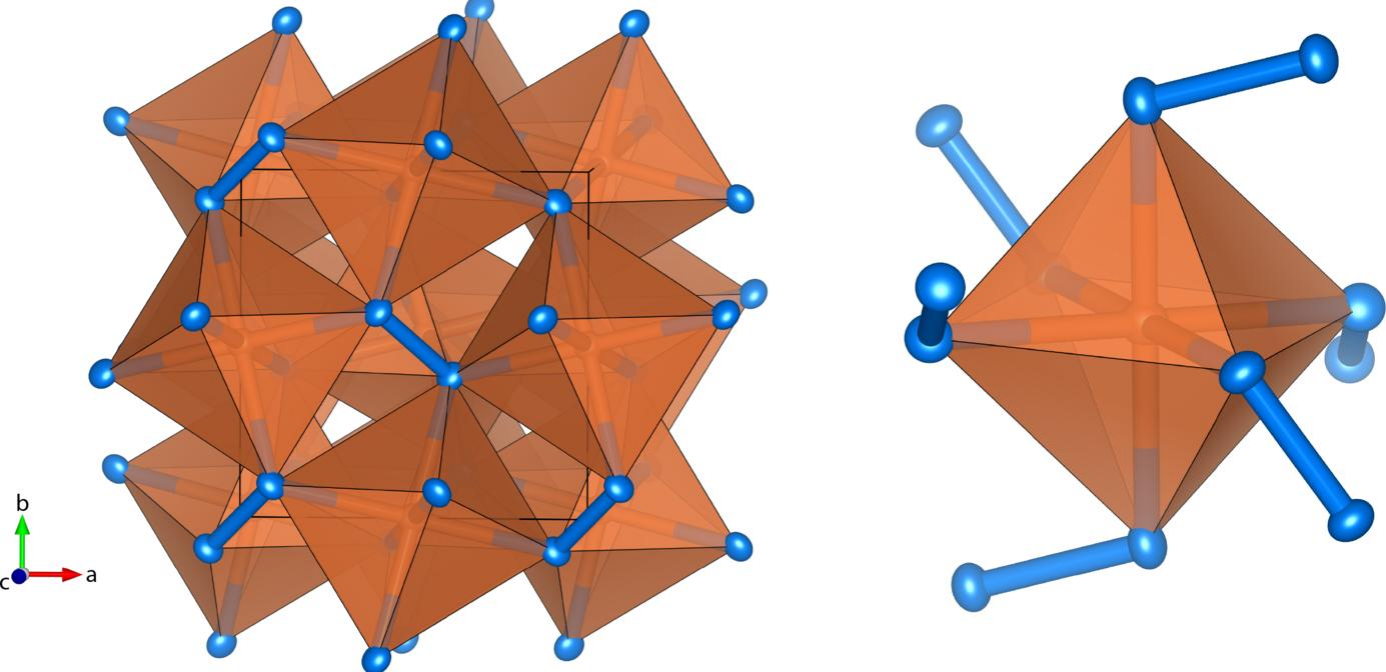
Crystal structure of SiN_2_ at 140 GPa in polyhedral representation with Si atoms in orange and N atoms in blue. Shown are [SiN_6_] octa­hedra inter­connected by N atoms. Displacement ellipsoids are represented at the 75% probability level.

SiN_2_ crystallizes in fact in the pyrite structure type in the space group *Pa* 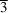 (No. 205). The asymmetric unit comprises two atoms, a silicon atom (multiplicity 4, Wyckoff letter *b*, site symmetry . 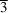 .), and a nitro­gen atom (8 *c*, .3.). The nitro­gen atoms form N—N dimers, and consequently each of the N atoms is tetra­hedrally coordinated by three Si atoms and one N atom. The centers of the N—N dimers form an *fcc* sublattice, which together with the inter­penetrating *fcc* sublattice of Si atoms can be considered as a derivative of the rock salt structure type. Slightly distorted [SiN_6_] octa­hedra [Si—N distance 6× 1.7517 (11) Å] inter­connect with each other by sharing common vertices (Fig. 1 ▸). There is a linear correlation between the nitro­gen–nitro­gen distance in dimers and the formal ionic charge and bond order of the (N_2_)^*x*−^ anion (Laniel *et al.*, 2022 ▸). In the case of SiN_2_, the refined nitro­gen–nitro­gen distance of 1.402 (8) Å indicates that the N—N bond has single-bond character. This distance is in a good agreement with N—N distances observed in other pernitrides that contain single-bonded (N—N)^4–^ units (Tasnádi *et al.*, 2021 ▸). However, it is longer compared to N—N bonds in diazenides (Laniel *et al.*, 2022 ▸; Bykov *et al.*, 2020 ▸) and in dinitrides of trivalent metals (Niwa *et al.*, 2014 ▸; Bykov *et al.*, 2019*b* ▸). Based on the empirical formula suggested by Laniel *et al.* (2022 ▸) for dinitrides, *FC* = (*BL* − 1.104)/0.074, where *FC* is the absolute value of the formal charge on the di­nitro­gen unit and *BL* is the N—N bond length in Å, a clear assignment can be made. For SiN_2_, the value of FC was calculated as 4.04, which is in excellent agreement with the most common oxidation state of +IV for silicon.

## 3. Synthesis and crystallization

A piece of silicon (10×10×5 µm^3^) was placed in the sample chamber of a BX90-type diamond anvil cell equipped with Boehler–Almax type diamonds using culets of 100 µm in diameter. The sample chamber was eventually created by laser-drilling a 50 µm hole in the Re gasket preindented to a thickness of 18 µm. Nitro­gen, loaded using the high-pressure gas-loading system of the Bavarian Geoinstitute (Kurnosov *et al.*, 2008 ▸), served both as a pressure-transmitting medium and as a reagent. Pressure was determined by the shift of the diamond Raman band (Akahama & Kawamura, 2006 ▸). Upon compression to the target pressure of 140 GPa, the sample was heated using a focused Nd:YAG laser (λ = 1064 nm) to temperatures exceeding 2500 K. The heating duration was approximately 10 seconds. The reaction products consisted of multiple high-quality, single-crystalline domains.

## 4. Refinement

Crystal data, data collection and structure refinement details are summarized in Table 1 ▸. The sample was studied by means of synchrotron single-crystal X-ray diffraction (SCXD) at the beamline ID11 (ESRF, Grenoble, France) with the following beamline setup: λ = 0.28457 Å, beamsize ∼0.7×0.7 *μ*m^2^, Eiger CdTe 2M detector. For the SCXD measurements, samples were rotated around a vertical ω-axis in the range ±30°. The diffraction images were acquired at an angular step Δω = 0.5° and an exposure time of 5 s per frame. For analysis of the single-crystal diffraction data (indexing, data integration, frame scaling and absorption correction) we used the *CrysAlis PRO* software package (Rigaku OD, 2023 ▸). To calibrate an instrumental mode using *CrysAlis PRO*, *i.e.*, the sample-to-detector distance, detector origin, offsets of goniometer angles, and rotation of both X-ray beam and the detector around the instrument axis, we used a single crystal of orthoenstatite [(Mg_1.93_Fe_0.06_)(Si_1.93_,Al_0.06_)O_6_, space group *Pbca*, *a* = 8.8117 (2), *b* = 5.18320 (10), and *c* = 18.239 (13) Å].

Data analysis followed several steps:

1. After collecting SCXD data sets (series of ∼120 frames), a 3D peak search procedure was performed as implemented *CrysAlis PRO*. This search identified reflections from all crystalline phases present in the collection spot, including reaction products, initial reagents, pressure-transmitting medium, diamonds and gasket material.

2. The peak search table was processed by means of the *DaFi* program (Aslandukov *et al.*, 2022 ▸), which sorts reflections into groups: if reflections fall into one group they origin­ate from one grain of the multigrain sample.

3. The reflection groups were assessed individually by indexing the reflections within the current group using built-in procedures in *CrysAlis PRO*. If indexing succeeded, the group was chosen for final data integration.

4. Datasets were integrated, and data was reduced following standard procedures, taking into account the shadowing of the diamond anvil cell.

## Supplementary Material

Crystal structure: contains datablock(s) I. DOI: 10.1107/S2056989023008058/wm5694sup1.cif

Structure factors: contains datablock(s) I. DOI: 10.1107/S2056989023008058/wm5694Isup2.hkl

CCDC reference: 2295150

Additional supporting information:  crystallographic information; 3D view; checkCIF report

## Figures and Tables

**Table 1:** Experimental details

Crystal data	
Chemical formula	SiN_2_
*M*_r_	56.11
Crystal system, space group	Cubic, *P* *a* 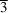
Temperature (K)	293
*a* (Å)	4.1205 (5)
*V* (Å^3^)	69.96 (3)
*Z*	4
Radiation type	Synchrotron, λ = 0.28457 Å
μ (mm^−1^)	0.21
Crystal size (mm)	0.001 × 0.001 × 0.001

Data collection	
Diffractometer	Customized ω-circle diffractometer
Absorption correction	Multi-scan (*CrysAlis PRO*; Rigaku OD, 2023 ▸)
*T*_min_, *T*_max_	0.750, 1.000
No. of measured, independent and observed [*I* > 2σ(*I*)] reflections	269, 101, 60
*R*_int_	0.049
(sin θ/λ)_max_ (Å^−1^)	1.112

Refinement	
*R*[*F* ^2^ > 2σ(*F* ^2^)], *wR*(*F* ^2^), *S*	0.071, 0.191, 1.07
No. of reflections	101
No. of parameters	6
Δρ_max_, Δρ_min_ (e Å^−3^)	1.08, −1.15

Computer programs: *CrysAlis PRO* (Rigaku OD, 2023 ▸), *SHELXT* (Sheldrick, 2015*a* ▸), *SHELXL* (Sheldrick, 2015*b* ▸), *VESTA* (Momma & Izumi, 2011 ▸) and *OLEX2* (Dolomanov *et al.*, 2009 ▸).

## supplementary crystallographic information

### Crystal data

**Table:**

SiN_2_	Synchrotron radiation, λ = 0.28457 Å
*M**_r_* = 56.11	Cell parameters from 79 reflections
Cubic, *P**a*3	θ = 3.4–16.4°
*a* = 4.1205 (5) Å	µ = 0.21 mm^−^^1^
*V* = 69.96 (3) Å^3^	*T* = 293 K
*Z* = 4	Irregular, colourless
*F*(000) = 112	0.001 × 0.001 × 0.001 mm
*D*_x_ = 5.327 Mg m^−^^3^	

### Data collection

**Table:**

Customized ω-circle diffractometer	101 independent reflections
Radiation source: synchrotron, ESRF, beamline ID11	60 reflections with *I* > 2σ(*I*)
Synchrotron monochromator	*R*_int_ = 0.049
Detector resolution: 5.0 pixels mm^-1^	θ_max_ = 18.5°, θ_min_ = 3.4°
ω scans	*h* = −4→4
Absorption correction: multi-scan (CrysAlisPro; Rigaku OD, 2023)	*k* = −7→8
*T*_min_ = 0.750, *T*_max_ = 1.000	*l* = −8→7
269 measured reflections	

### Refinement

**Table:**

Refinement on *F*^2^	0 restraints
Least-squares matrix: full	Primary atom site location: dual
*R*[*F*^2^ > 2σ(*F*^2^)] = 0.071	*w* = 1/[σ^2^(*F*_o_^2^) + (0.109*P*)^2^] where *P* = (*F*_o_^2^ + 2*F*_c_^2^)/3
*wR*(*F*^2^) = 0.191	(Δ/σ)_max_ < 0.001
*S* = 1.07	Δρ_max_ = 1.08 e Å^−^^3^
101 reflections	Δρ_min_ = −1.15 e Å^−^^3^
6 parameters	

### Special details

**Table:**

Geometry. All esds (except the esd in the dihedral angle between two l.s. planes) are estimated using the full covariance matrix. The cell esds are taken into account individually in the estimation of esds in distances, angles and torsion angles; correlations between esds in cell parameters are only used when they are defined by crystal symmetry. An approximate (isotropic) treatment of cell esds is used for estimating esds involving l.s. planes.

### Fractional atomic coordinates and isotropic or equivalent isotropic displacement parameters (Å^2^)

**Table:**

	*x*	*y*	*z*	*U*_iso_*/*U*_eq_	
Si1	0.5000	0.5000	0.5000	0.0084 (5)	
N1	0.5982 (5)	0.4018 (5)	0.9018 (5)	0.0076 (6)	

### Atomic displacement parameters (Å^2^)

**Table:**

	*U*^11^	*U*^22^	*U*^33^	*U*^12^	*U*^13^	*U*^23^
Si1	0.0084 (5)	0.0084 (5)	0.0084 (5)	−0.0005 (3)	−0.0005 (3)	−0.0005 (3)
N1	0.0076 (6)	0.0076 (6)	0.0076 (6)	0.0012 (8)	0.0012 (8)	−0.0012 (8)

### Geometric parameters (Å, º)

**Table:**

Si1—N1	1.7517 (11)	Si1—N1^v^	1.7517 (11)
Si1—N1^i^	1.7517 (11)	N1—Si1^vi^	1.7517 (11)
Si1—N1^ii^	1.7517 (11)	N1—Si1^vii^	1.7517 (11)
Si1—N1^iii^	1.7517 (11)	N1—N1^viii^	1.402 (8)
Si1—N1^iv^	1.7517 (11)		
			
N1—Si1—N1^iii^	86.94 (4)	N1^iv^—Si1—N1^ii^	86.94 (4)
N1^iv^—Si1—N1^v^	93.06 (4)	N1^v^—Si1—N1^i^	86.94 (4)
N1^ii^—Si1—N1^i^	93.06 (4)	N1—Si1—N1^v^	86.94 (4)
N1—Si1—N1^iv^	180.0	N1—Si1—N1^i^	93.06 (4)
N1^iii^—Si1—N1^i^	180.00 (14)	Si1—N1—Si1^vi^	112.54 (10)
N1^iii^—Si1—N1^iv^	93.06 (4)	Si1^vii^—N1—Si1^vi^	112.54 (10)
N1^iii^—Si1—N1^v^	93.06 (4)	Si1^vii^—N1—Si1	112.54 (10)
N1—Si1—N1^ii^	93.06 (4)	N1^viii^—N1—Si1^vi^	106.20 (12)
N1^ii^—Si1—N1^v^	180.0	N1^viii^—N1—Si1^vii^	106.20 (12)
N1^iii^—Si1—N1^ii^	86.94 (4)	N1^viii^—N1—Si1	106.20 (12)
N1^iv^—Si1—N1^i^	86.94 (4)		
			
N1^iii^—Si1—N1—Si1^vii^	94.59 (6)	N1^i^—Si1—N1—Si1^vii^	−85.41 (6)
N1^iii^—Si1—N1—Si1^vi^	−33.8 (3)	N1^ii^—Si1—N1—Si1^vii^	7.82 (11)
N1^v^—Si1—N1—Si1^vi^	59.4 (2)	N1^iii^—Si1—N1—N1^viii^	−149.62 (10)
N1^i^—Si1—N1—Si1^vi^	146.2 (3)	N1^v^—Si1—N1—N1^viii^	−56.39 (6)
N1^ii^—Si1—N1—Si1^vi^	−120.6 (2)	N1^i^—Si1—N1—N1^viii^	30.38 (10)
N1^v^—Si1—N1—Si1^vii^	−172.18 (11)	N1^ii^—Si1—N1—N1^viii^	123.61 (6)

Symmetry codes: (i) −*x*+1, *y*+1/2, −*z*+3/2; (ii) −*x*+3/2, −*y*+1, *z*−1/2; (iii) *x*, −*y*+1/2, *z*−1/2; (iv) −*x*+1, −*y*+1, −*z*+1; (v) *x*−1/2, *y*, −*z*+3/2; (vi) −*x*+1, *y*−1/2, −*z*+3/2; (vii) −*x*+3/2, −*y*+1, *z*+1/2; (viii) −*x*+1, −*y*+1, −*z*+2.
